# Primary health institutions preference by hypertensive patients: effect of distance, trust and quality of management in the rural Heilongjiang province of China

**DOI:** 10.1186/s12913-019-4465-7

**Published:** 2019-11-20

**Authors:** Jingjing Liu, Hui Yin, Tong Zheng, Bykov Ilia, Xing Wang, Ruohui Chen, Yanhua Hao, Hong Sun, Mingli Jiao, Zheng Kang, Lijun Gao, Qunhong Wu

**Affiliations:** 1 Department of Social Medicine, School of Public Health, Harbin Medical University, 157 Baojian Road, Nangang District, Harbin, 150081 China; 2 Department of health education, School of Public Health, Harbin Medical University, 157 Baojian Road, Nangang District, Harbin, 150081 China

**Keywords:** Hospital preference, Primary health institutions, Hypertension management, Doctor-patient trust

## Abstract

**Background:**

Traditional “inverted triangle” healthcare resources allocation model in China has wasted a lot of health resources. The Chinese health reform began to strengthens the role of the primary health institutions in delivering primary health care especially in rural areas in the background of large development gap between urban-rural health and rapid growth in the incidence of chronic diseases in rural. We take hypertensive patients as an example, to verify the effect of policy implementation through distribution characteristics of rural primary health institutions preference of hypertensive patients and explore the influencing factor that promoting rationalized use of medical care for patients with chronic disease as well as rational allocation of health resources in rural areas.

**Methods:**

A cross-sectional survey was conducted in Heilongjiang, a province in northeastern China by using a self-designed questionnaire. Stratified cluster sampling was used to choose 484 hypertensive patients from two villages in Heilongjiang province in 2010.

**Results:**

About 88.4% of respondents reported preferred primary health institutions (83.5% preferred village clinics and 4.9% preferred township hospitals), 49.4% of respondents knew hypertension management administered by primary health institutions, 53.5% received hypertension education from primary care physicians, more than half of respondents reported that they didn’t receive telephone interviews and family visits from primary care physicians over the past 6 months. Residence closer to the primary health institutions (OR = 10.360), trust in village doctors (OR = 7.323), elders (OR = 3.001), and asked for return visits by primary health physicians (OR = 2.073) promote preferences for primary health institutions.

**Conclusions:** Accessibility to primary healthcare and doctor-patient trust stimulate patients to choose the primary health institutions. Primary health institutions should improve general approach to hypertension management and enhance the ability of providing basic public health services.

**Electronic supplementary material:**

The online version of this article (10.1186/s12913-019-4465-7) contains supplementary material, which is available to authorized users.

## Background

Traditional healthcare model in China is shaped like an “inverted triangle”, with most loaded large hospitals on the top, following by medium-sized hospitals and primary health institutions on the bottom. Such patient flow tendency of medical visits lead to higher costs of health services [1, 2]. And that is the opposite to international advocacy, which primary health institutions should act as the health gatekeepers for patients, especially with chronic disease and conditions such as diabetes, cardiovascular disease and cancer which are the leading causes of morbidity and mortality worldwide [3]. Evidences had proven that morbidity and mortality cause by chronic conditions could be reduced only by coordinated public health prevention policies in primary health institutions [4]. The strong and effective primary health care eliminate healthcare disparities, reduce cost and improve quality of medical services, specialized and inpatient care [5–7]. China’s 2009 health reforms emphasize focusing on rural areas and reconstruction of healthcare to improve service capacity and condition of rural primary health institutions, including village clinics and township hospitals. The reforms advocate that major part of villagers’ health problems could be solved without going out of the countryside, by establishing of a 15-min health service circle in rural areas [8].

Chronic disease prevention and management were identified as a high public health need in the China’s 2009 health reforms [8]. China has the biggest number of patients with hypertension in the world [9, 10] and 50.3% of whom reside in rural areas [11]. There are some problems in rural areas such as low level of healthcare services and poor accessibility to healthcare [12]. To eliminate the urban-rural disparity, the rural medical services system had been vigorously developed, along with the rural three-tiered health service network, which consists of country hospitals (which focus on treating serious conditions), township hospitals (common disorders), and village clinics (minor illnesses). Rural primary health institutions (township hospitals and village clinics) are the main providers of basic public health services and they are also centers of hypertension management in those areas. Primary care physicians are required to measure blood pressure, register patients, follow-up of them, and provide hypertension education [13, 14].

In the background of improving rural health, we will verify the effect of policy implementation through flow tendency of medical visits of rural patients and hypertension management in rural primary health institutions especially the remote areas of China. So we decided to conduct the study in the rural area of the northeast region in China where recorded the highest incidence of hypertension, [15, 16] to explore factors associated with primary care institutions preference and current status of hypertension management in primary health institutions of high latitude and mountainous region. The findings of such study can provide evidence and support for the rational distribution of healthcare resources in rural area, in order to rationalize medical care for patients with hypertension in these areas, improve treatment providing by primary care physicians and their relationship with those patients.

## Methods

### Study design, sample and populations

Undergraduate medical students conducted face-to-face interviews in village clinics and administration offices, from July 23 to 26, 2010. All interviewers received training and practiced an interview before conducting an actual one. The original version of the questionnaire was distributed among 25 people, on purpose to evaluate feedback and revised its content if necessary (Additional file 1).

Hypertensive patients were invited to participate in a survey distributed by village doctors. Interviewers clearly explained the purposes and confidentiality of the survey before inviting patients to participate in the interview or proceeding with the interview. The participation of respondents in the interview was voluntary and informed consent was obtained from respondents. During the whole process, interviewers measured participant’s blood pressure and collected data using the questionnaire guide. The completeness of the interviews was checked right after the survey had been completed. The interviewers contacted participants by telephone to fill the missing information in, if there was a necessity.

A cross-sectional face-to-face survey of hypertensive patients in the rural areas of Heilongjiang province in the northeast China was conducted. To obtain the two convenience samples, two counties, Fujin and Linkou, which were willing to cooperate with the survey and had convenient transportation access for this study’s surveyors, were selected. The classification standard for categorizing towns within the two counties into groups was economic development status (i.e., relatively poor and non-poor). Two convenient towns were selected from each economic development status. Then in selected townships, all villages with at least 800 inhabitants were classified into low, middle, and high economic development group and two accessible for sampling villages were selected out of each group. In total 24 villages were picked for the study.

The determining criteria of the economic development were defined by the staff of the countries’ health departments. Participants for this study were picked from the existing registries of the patients with hypertension. Generally, there is at least one physician who is responsible for conducting regular physical checkups to residents of the each village. In case of hypertension, to diagnose such condition physicians are using the criteria of the Joint National Committee on High Blood Pressure-7 (JNC-7) – SBP > 140 or DBP > 90 mmHg, or current use of antihypertensive drugs. Village physicians are paid by fee-for-service scheme and they motivated to register and manage as much patients as possible, thus the registry is relatively complete and frequently updated. The researchers met with the physicians to determine the selection criteria for participation in the study. Due to fact that physicians are not only registering patients but also residing in the village, so they know the patients pretty well. In addition, patients with secondary hypertension, people living with dementia, severe mental disorder, or slurred speech were excluded.

Hypertensive patients preferred type of institution was considered valid for analysis, if in the previous 6 months patient showed a health-seeking behavior or received any medical treatment because of hypertension. Among the 900 hypertension participants, 509 received medical treatment because of hypertension in the past 6 months and invited these people to participate in the survey. Excluding the questionnaire that failed to meet eligibility criteria, a total of 484 hypertension 119 patients participated in the study (response rate: 95.09%). The informed consents were accepted and signed by all participants of the study. The Ethics Committee of Harbin Medical University approved the study.

### Measures

To determine patients’ preferences, they were asked to choose in which type of hospitals they prefer to receive a medical care - “village clinics”, “township hospitals”, “county hospitals” or “municipal hospitals and higher-level hospitals”. For performing the logistic regression modeling, facility types were divided into two groups: “primary health institutions” (including “village clinics” and “township hospitals”) and “non-primary health institutions” (including “county hospitals” and “higher-level hospitals”).

Independent variables included in the modeling contained the following five modules with their contents:

1. Socio-demographic characteristics: Gender, age, education and insurance status.
2. Self-rated physical health: That was determined by asking the participants an open-question. “How do you evaluate your health?”. Possible answers were “well”, “moderate” and “poor”.
3. The nearest health institutions: That was determined by asking the participants an open-question. “What is the nearest medical institution from to your home?”. Possible answers were “village clinics”, “township hospitals” and “county hospitals and higher-class hospitals”. They were divided into two groups: “primary health institutions” (including “village clinics” and “township hospitals”) and “non-primary health institutions” (including “county hospitals” and “higher-level hospitals”) for the modeling.
4. Subjective evaluation of rural primary care physicians: This module contained the following four questions (Table 1). The level of satisfaction was initially measured using a 5-point Likert scale (complete dissatisfaction; dissatisfaction; neutral; satisfaction, complete satisfaction). These were divided into two groups: “satisfaction” (complete satisfaction, satisfaction, neutral) and “dissatisfaction” (complete dissatisfaction and dissatisfaction). The level of trust was also initially measured using a 5-point Likert scale (complete distrust; distrust; neutral; trust, complete trust). These were divided into two groups: “trust” (complete trust, trust and neutral) and “distrust” (complete distrust and distrust) for the modeling (Table 1).
5. Status of hypertension management in primary health institutions: This module contained the following five questions which could be answered with a “yes” or a “no” (Table 1).

**Table 1:** Questions and possible answers for rural primary health care system and current status of hypertension management in primary health institutions

Questions	Answers
*Subjective evaluation of rural primary care physicians*	
① “Do you satisfied with disease prevention carrying out by village doctors?”	complete dissatisfaction; dissatisfaction; neutral; satisfaction; complete satisfaction
② “Do you satisfied with medical services carrying out by village doctors?”	complete dissatisfaction; dissatisfaction; neutral; satisfaction; complete satisfaction
③ “Do you trust in village doctors?”	complete distrust; distrust; neutral; trust; complete trust
④“Do you trust in doctors in township hospitals?”.	complete distrust; distrust; neutral; trust; complete trust
*Status of hypertension management in primary health institutions*	
① “Did you know that primary care institutions must control your hypertension treatment?”	“yes” or “no”
② “Did you receive hypertension education from primary care physicians over the past 6 months?”	“yes” or “no”
③ “Was you interviewed via telephone by primary care physicians over the past 6 months?”	“yes” or “no”
④ “Did primary care physicians perform family visits to your over the past 6 months?”	“yes” or “no”
⑤ “Was you asked for return visits by primary care physicians over the past 6 months?”.	“yes” or “no”

### Statistical analysis

Data analyses were performed with SPSS 21.0 (IBM Corp, Armonk, USA). The outcome variable was hospitals preferences dichotomized into primary health institutions preference and non-primary health institutions preference. For bivariate analysis of categorical variables we used chi square test. Independent variables included were socio-demographic characteristics factors, physical health factors, the distance factors, subjective evaluation of rural primary care physicians factors and status of hypertension management in primary health institutions factors. We calculated Pearson’s correlation coefficients to rule out multicollinearity between predictor variables. We used multivariate logistic regression models in which factors were found to be significant (*p* < 0.05) were added step-by-step. Adjusted Odds Ratios and their 95% CI were calculated. A *p*-value less than 0.05 was considered as significant.

## Results

Of the 484 respondents, 88.4% of respondents preferred primary health institutions (83.5% preferred village clinics and 4.9% preferred township hospitals) (Table 2).

**Table 2:** The distribution of hospital preference of rural hypertensive patients (*N* = 484)

Hospital preference	Primary health institutions preference				Non-primary health institution preference			
	Village clinics		Township hospitals		County hospitals		Municipal hospitals and higher-level hospitals	
N (% of 484)	n	Percent	n	Percent	n	Percent	N	Percent
	404	83.5	24	4.9	43	8.9	13	2.7

More than half of all respondents (63%) were men, 28.7% were over the age of 65 and only 3.1% had education above junior high school at the time of the survey. Chi-square tests revealed that primary health institutions preference was associated with age, education, the nearest health institution, satisfaction with disease prevention carrying out by village doctors, satisfaction with medical services carrying out by village doctors, trust in village doctors and being asked for return visits by primary care physicians (*P* < 0.05) (Table 3).

**Table 3:** Socio-demographic characteristics of respondents and primary health institutions preference outcomes (*N* = 484)

Variables	*N* (% of 484)	Primary health institutions preference *n* (% of 428)	Non-primary health institution preference *n* (% of 56)	*p*-Value
Gender				0.932
Male	179 (37.0)	158 (32.6)	21 (4.4)	
Female	305 (63.0)	270 (55.8)	35 (7.2)	
Age				0.038
< =55	156 (32.2)	130 (26.9)	26 (5.4)	
55–65	204 (42.1)	183 (37.8)	21 (4.3)	
> =65	124 (25.7)	115 (23.8)	9 (1.8)	
Education				0.083
Junior high school education and below	469 (96.9)	417 (86.2)	52 (10.7)	
Above junior high school education	15 (3.1)	11 (2.3)	3 (0.8)	
Insurance status				0.114
No social health insurance	39 (8.1)	34 (7.0)	5 (1.0)	
New Cooperative Medical Scheme	426 (88.0)	380 (78.5)	46 (9.5)	
Other social health insurance^a^	19 (3.9)	14 (3.0)	5 (1.0)	
Self-rated physical health				0.834
Well	245 (50.6)	217 (44.8)	28 (5.8)	
Moderate	157 (32.4)	140 (28.9)	17 (3.5)	
Poor	82 (17.0)	71 (14.7)	11 (2.3)	
The nearest health institutions				0.004
Primary health institutions	7 (1.45)	3 (0.8)	4 (0.8)	
Non-primary health institution	477 (98.55)	425 (87.8)	52 (10.6)	
Satisfaction with disease prevention carrying out by village doctors				0.002
Satisfaction	462 (95.5)	413 (85.3)	49 (10.1)	
Dissatisfaction	22 (4.5)	15 (3.1)	7 (1.5)	
Satisfaction with medical services carrying out by village doctors				0.001
Satisfaction	472 (97.5)	421 (87.0)	51 (10.5)	
Dissatisfaction	12 (2.5)	7 (1.5)	5 (1.0)	
Trust in village doctors				0.000
Trust	471 (97.3)	421 (87.0)	50 (10.3)	
Distrust	13 (2.7)	7 (1.5)	6 (1.2)	
Trust in doctors in township hospitals				0.113
Trust	346 (71.5)	311 (64.3)	35 (7.2)	
Distrust	138 (28.5)	117 (24.2)	21 (4.3)	
*Current status of hypertension management in primary health institutions*				
Knew that their hypertension treatment must be controlled by primary care institutions				0.108
Yes	239 (49.4)	217 (44.8)	22 (4.5)	
No	245 (50.6)	211 (43.6)	34 (7.1)	
Received hypertension education from primary care physicians over the past 6 months				0.152
Yes	259 (53.5)	224 (46.3)	35 (7.2)	
No	225 (46.5)	204 (42.2)	21 (4.3)	
Received telephone interviews from primary care physicians over the past 6 months				0.409
Yes	153 (31.6)	138 (28.5)	41 (8.5)	
No	331 (68.4)	290 (59.9)	15 (3.1)	
Received family visits from primary care physicians over the past 6 months				0.073
Yes	164 (33.9)	151 (31.2)	43 (8.9)	
No	320 (66.1)	277 (57.2)	13 (2.7)	
Being asked for return visits by primary care physicians over the past 6 months				0.012
Yes	249 (51.4)	229 (47.3)	20 (4.1)	
No	235 (48.6)	199 (41.1)	36 (7.5)	

^a^ Other social health insurance includes Medical Insurance for Urban Employees (MIUE), Medical Insurance for Urban Residents (MIUR), Full Public Expense, and Medical Insurance

Those independent variables that were confirmed to be statistically significant by Chi-square tests were entered into the logistic regression model. Respondents whose places of residence are closer to the primary health institutions were about 10 times (OR = 10.360, 95% CI 2.090 to 51.343; *P* < 0.01) more likely to prefer primary health institutions over others than those whose places of residence are closer to county hospitals and higher-level hospitals. In comparison with the respondents who distrust in village doctors, respondents which trust (OR = 7.323, 95% CI 2.292 to 23.399; *P* < 0.001) were more likely to prefer primary health institutions. Age (OR = 3.001, 95% CI 1.287 to 6.999; *P* < 0.05) were also significant predictors for primary health institutions preference. We also founded out that those who were asked for return visits by primary health physicians over the past 6 months were about two times (OR = 2.073, 95% CI 1.128 to 3.808; P < 0.05) more likely to prefer primary health institutions over others than those who weren’t asked for return visits (Table 4).

**Table 4:** Factors associated with primary health institutions preference

Variables	B	Walds	P	OR	95% CI	
Age group (55–65) vs (<=55)	0.329	3.479	0.062	1.847	0.969	3.520
Age group (> = 65) vs (<=55)	0.432	6.472	0.011	3.001	1.287	6.999
The nearest health institutions (primary health institutions vs non-primary health institution)	0.817	8.196	0.004	10.360	2.090	51.343
Trust in village doctors vs distrust	0.593	11.282	0.001	7.323	2.292	23.399
Being asked for return visits by primary care physicians over the past 6 months vs not being asked	0.310	5.516	0.019	2.073	1.128	3.808

**Fig. 1:**
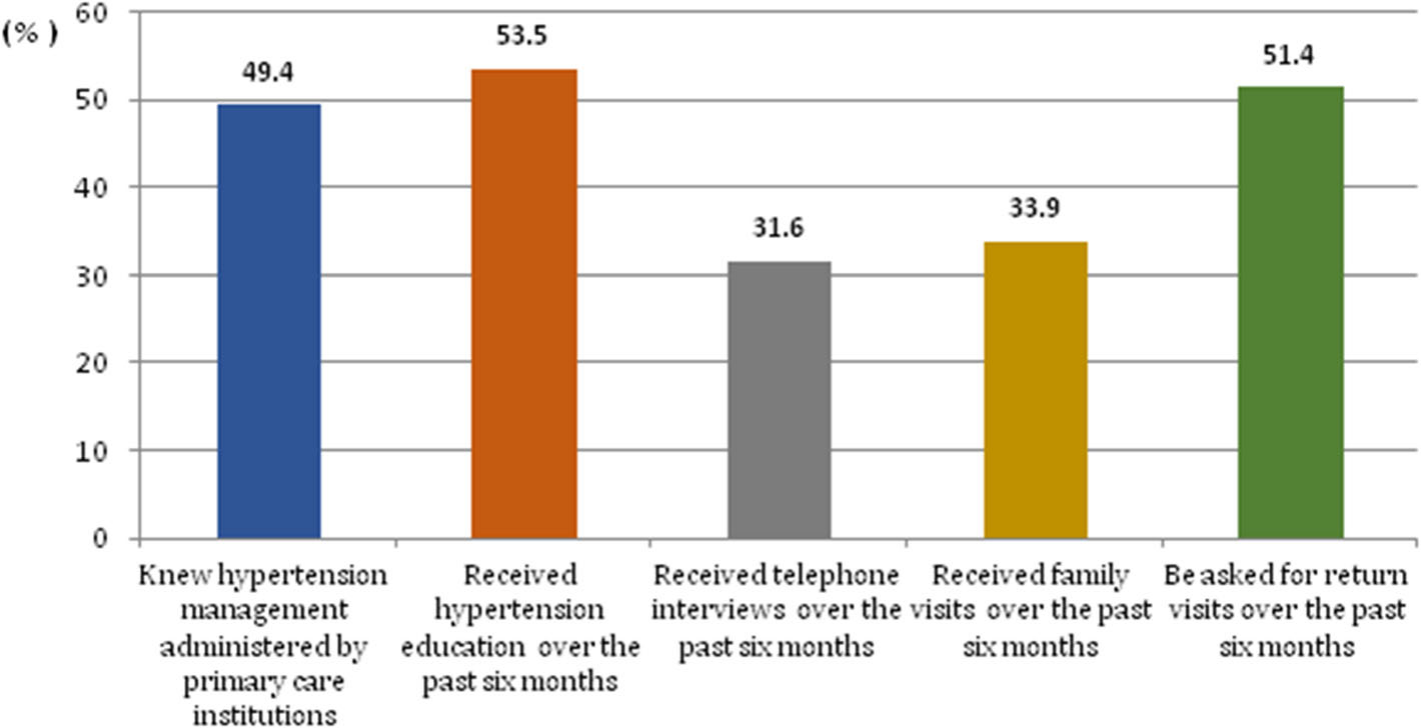
is described using the column chart. The vertical axis indicates the proportion of the number of people who’s perception about current status of hypertension management in primary health institutions among all respondents. The horizontal axis represents patient perception about current status of hypertension management in primary health institutions. Different color columns represent different perception, as shown below. Showed that knew that primary care institutions must control your hypertension treatment. Showed that received hypertension education over the past 6 months. Showed that received telephone interviewed over the past 6 months. Showed that received family visits over the past 6 months. Showed be asked for a return visits over the past 6 months

Among respondents, 49.4% knew that their hypertension treatment must be controlled by primary health institutions and 53.5% received hypertension education from primary care physicians over the past 6 months. More than a half of respondents reported that they didn’t receive telephone interviews and family visits from primary care physicians over the past 6 months. 51.4% of respondents stated that they were asked for return visits to primary health physicians over the past 6 months (Fig. 1).

This study focused on the primary health institutions preference of rural hypertensive patients in China. The results showed that 88.4% of the respondents preferred primary health institutions while only 11.6% preferred higher-level medical institutions. These results are meeting the statement set by the World Health Organization that “more than 80% of the health problems of residents can be solved at primary health care facilities”. Such a high proportion of preference for primary health institutions had also been found in studies undertaken elsewhere in China [17, 18]. Closer distance, trust between doctor-patient, elders, asked for return visits promote preferences for primary health institutions. However, as a major department of hypertension management, primary health institutions are not well managed.

Distance to the nearest medical institutions was identified as the most important factor that have the strongest association with primary health institutions preference. Among those who preferred primary health institutions, 86.6% showed that village clinics are the nearest medical institutions to their place of residence. And the Respondents whose places of residence are closer to the village clinics were about 10 times (OR = 10.360) more likely to prefer primary health institutions over others than those than whose places of residence are closer to county hospitals and higher-level hospitals. This result is consistent with previous studies [19–23]. Peter proposed that travel time is much more important as a determinant of hospital choice [19]. Robert et al. also found that distance is a deterrent factor for choice of concrete hospitals [23]. Akinci et al. highlighted the importance of accessibility of hospital services to the population [ 24]. Reorganization of the village clinics is an important measure to improve accessibility of primary healthcare to the patients in order to provide them affordable medical services which are convenient for consuming. As a part of the China’s 2009 nationwide health reforms, rural primary health institutions were proclaimed a health care gatekeepers for a rural population, that along with using of the former village medical units, such as health centers and medical point, were intended to ensure better, cheaper, more convenient and safer medical services for patients in rural areas.

Trust remains an important determinant for patients in making a choice on the type of hospital. In our study, we found that in comparison with the respondents who distrust in village doctors, respondents which trust (OR = 7.323) were more likely to prefer primary health institutions..Patient trust increases uptake of, engagement with and optimal outcomes from healthcare services. [25] Mechanic argued that interpersonal trust depends on the degree to which patients see their doctors as competent, responsible, and caring. [26] Higher level of education to applicants for the positions and performance appraisal (PA) system were gradually applied to primary health physicians selection and evaluation in order to improve technical and standard treatment behavior [ 27–29]. If physicians pay attention to profits and are seen by the public as being profit-driven, they are likely to induce mistrust from patients [30]. Drugs in primary health institutions are sold with zero markup and the profit is subsidized by the government to eliminate an opportunity for primary health physicians to get profit from patients. This is a characteristic phenomenon in China that most village doctors and villagers live in the same familiar village and in the social environment of acquaintances in the village, the relationship between village doctors and villagers is not only limited to the doctor-patient relationship caused by medical treatment, but also the unique interpersonal emotional relationship generated by frequent contact and long-term relationship.

The survey found that being asked for return visits by primary health physicians over the past 6 months strengthened the will of patients to choose primary health institutions over others. It’s a successful practice of hypertension management. However, the survey showed the overall situation of hypertension management in rural areas is not so optimistic: low awareness rate about primary health institutions control functions in management of hypertension (49.4%), low coverage rate of health education within hypertensive patients (53.5%) and low regular follow-up rate (< 35%) over the past 6 months. Current situation may be caused by the following reasons. First, supportive policy environment has not been formed yet for example, limit administrative privileges, poor sectoral cooperation and lack of funds, result in lack of agitation and insufficient adherence to standards in management of hypertension, so society has insufficient understanding on importance of proper hypertension management, what leads to poor patients’ compliance. Secondly, inadequate construction of monitoring mechanism that lacks effective assessment system for chronic disease monitoring and data standardization. Chronic disease management is provided only for high-risk groups and has narrow and insufficient services coverage. It is necessary to pay attention to the quality control. Thirdly, lack of professional public health personnel along with their poor clinical knowledge and insufficient competence on matters of prevention and management of hypertension lead to following inadequate follow-up management.

## Limitation

This study existed limitations. Since we had investigated situation only in two villages of Heilongjiang province located at mountainous and high latitude region, which may not represent the real overall situation within a whole target population, so these findings can’t be generalized to other geographic areas of China. Secondly, data were collected retrospectively; this method depends on the ability of participants to recall events that had occurred within 6 months before our study, potentially resulting in recall bias. Thirdly, primary health institutions preference results may be overestimated because of using self-assessment tools. Besides, this survey has been conducted several years ago; some result might not fully capture the recent reform changes. However, the exploration of factors affecting patients’ preferences might be valuable for improving service capacity of primary health institutions.

## Conclusion

Accessibility to primary healthcare and doctor-patient trust are important factors affecting hypertensive patients’ preferred primary health institutions in rural areas but primary health institutions showed an insufficient adherence to standards in management of hypertension. The findings of this study and causes analysis provided necessary evidence for strengthening the management of hypertension in rural primary health institutions, rational allocation of rural healthcare resources, enhancing the primary health institutions’ ability to provide basic public health services and also, for promotion of the flow of medical treatment for rural patients is more reasonable.

## Additional file

Additional file 1: Hypertension patient questionnaire. (DOC 41 kb)

## Data Availability

Data are available from the corresponding author upon request.

## Abbreviations

CI: Confidence interval
DBP: Diastole Blood Pressure
JNC-7: The Seventh Report of the Joint National Committee on the Prevention, Detection, Evaluation, and Treatment of High Blood Pressure
OR: Odds Ratios
PA: Performance appraisal
SBP: Systolic Blood Pressure
