# Supplementary material for: Primary health institutions preference by hypertensive patients: effect of distance, trust and quality of management in the rural Heilongjiang province of China
Source: BMC Health Serv Res. 2019 Nov 20;19:852. doi: 10.1186/s12913-019-4465-7 (PMC6868842; doi:10.1186/s12913-019-4465-7)
Supplement: Supplementary file 1 — Hypertension patient questionnaire. (DOC 41 kb) [file 12913_2019_4465_MOESM1_ESM.doc]

**Hypertension patient questionnaire**

1. Age: (years)

2. Gender：

① male ② female

3. Education:

① illiteracy ② primary school ③middle school ④high school and above

4. Insurance status:

① No social health insurance

② New Cooperative Medical Scheme

③ Medical Insurance for Urban Employees (MIUE)

④ Medical Insurance for Urban Residents (MIUR)

⑤ Full Public Expense

⑥ Medical Insurance

5. Self-rated physical health:

① well ② moderate ③ poor

6. What is the nearest medical institution from to your home？

① village clinics

② township hospitals

③ county hospitals and higher-class hospitals

7. Do you satisfied with disease prevention carrying out by village doctors？

① complete dissatisfaction

② dissatisfaction

③ neutral

④ satisfaction

⑤ complete satisfaction

8. Do you satisfied with medical services carrying out by village doctors？

① complete dissatisfaction

② dissatisfaction

③ neutral

④ satisfaction

⑤ complete satisfaction

9. Do you trust in village doctors?

① complete distrust

② distrust

③ neutral

④ trust

⑤ complete trust

10. Do you trust in doctors in township hospitals?

① complete distrust

② distrust

③ neutral

④ trust

⑤ complete trust

11. Did you know that primary care institutions must control your hypertension treatment?

①yes

②no

12. Did you receive hypertension education from primary care physicians over the past six months?

①yes

②no

13. Was you interviewed via telephone by primary care physicians over the past six months?

①yes

②no

14. Did primary care physicians perform family visits to your over the past six months?

①yes

②no

15. Was you asked for return visits by primary care physicians over the past six months?

①yes

②no
